# Climate Change, Carbon Dioxide Emissions, and Medical Imaging Contribution

**DOI:** 10.3390/jcm12010215

**Published:** 2022-12-27

**Authors:** Eugenio Picano, Cristina Mangia, Antonello D’Andrea

**Affiliations:** 1 Institute of Clinical Physiology, CNR, 56124 Pisa, Italy; 2 ISAC—Institute of Sciences of Atmosphere and Climate, CNR, 73100 Lecce, Italy; 3 Cardiology Division, Nocera Inferiore Hospital, Nocera Inferiore, 84014 Salerno, Italy

**Keywords:** environment, imaging, planet, sustainability

## Abstract

Human activities have raised the atmosphere’s carbon dioxide (CO_2_) content by 50% in less than 200 years and by 10% in the last 15 years. Climate change is a great threat and presents a unique opportunity to protect cardiovascular health in the next decades. CO_2_ equivalent emission is the most convenient unit for measuring the greenhouse gas footprint corresponding to ecological cost. Medical imaging contributes significantly to the CO_2_ emissions responsible for climate change, yet current medical guidelines ignore the carbon cost. Among the common cardiac imaging techniques, CO_2_ emissions are lowest for transthoracic echocardiography (0.5–2 kg per exam), increase 10-fold for cardiac computed tomography angiography, and 100-fold for cardiac magnetic resonance. A conservative estimate of 10 billion medical examinations per year worldwide implies that medical imaging accounts for approximately 1% of the overall carbon footprint. In 2016, CO_2_ emissions from magnetic resonance imaging and computed tomography, calculated in 120 countries, accounted for 0.77% of global emissions. A significant portion of global greenhouse gas emissions is attributed to health care, which ranges from 4% in the United Kingdom to 10% in the United States. Assessment of carbon cost should be a part of the cost-benefit balance in medical imaging.

## 1. Introduction

In the present paper, we present an opinion piece on the environmental impact of medical imaging, with a special focus on cardiac imaging, and the possible ways to reduce it. We focus on cardiac imaging for three reasons. First, cardiac imaging examinations are one of the most important applications of medical imaging (>50% of all nuclear medicine and invasive fluoroscopy examinations) and are rising with the advent of coronary computed tomography (CT) [1]. Second, the relevance of cardiac imaging is especially timely since the recent European Society of Cardiology, American College of Cardiology, and American Heart Association general cardiology guidelines established the equipoise of five different imaging techniques for the diagnosis of chest pain but made no mention of the environmental impact of the imaging options. This included stress with ultrasound (US), CT, stress with magnetic resonance imaging (MRI), and nuclear techniques such as stress with positron emission tomography (PET) and stress with single-photon emission tomography (SPECT) [2,3]. Third, as cardiologists and imagers, we are aware that the culture of environmental sustainability has been present in radiology culture and practice for years [4] but ignored by mainstream cardiology culture. 

## 2. Climate Changes and Carbon Dioxide 

The global influence of the environment profoundly affects cardiovascular risk, incidence, prevalence, and severity [5]. Environmental stimuli include social stress, diet, smoking, chemical contaminants, noise, sleep disturbances, ionizing radiation, air pollution, and climate change [6]. 

Global warming is altering the earth’s global landscape and ocean temperatures. To date, an increase of 1.09 °C has been observed in recent years compared to pre-industrial levels. Each of the last four decades has been progressively warmer than any previous decade since 1850. Consequently, temperature variability, heat waves, forest fires, desert storms, and extreme cold events have become more frequent in many parts of the world, and the number of deaths increases significantly with the repetition of extreme events [7]. The Lancet Countdown on Health and Climate Change reported the effects of extreme temperatures on health and disease, including cardiovascular diseases. A 2021 global analysis estimated that more than 5 million deaths per year are associated with non-optimal temperatures [8]. By modifying weather patterns and the consequent emission scenarios, global warming leads to worsening air pollution, which in turn influences climate change. Although they are distinct phenomena occurring at different spatiotemporal scales, the two environmental crises are considered two sides of the same coin [9,10]. These trends are expected to worsen in the coming years in the absence of effective countermeasures [11]. 

It is widely accepted that climate change is caused by high levels of greenhouse gas (GHG) emissions, such as carbon dioxide (CO_2_), methane, nitrous oxide, etc., due to human activity [11]. From 2010–2019 the average annual global GHG emissions were at their highest levels in human history. Human activities have raised the atmosphere’s CO_2_ content by 50% in less than 200 years and by 10% in the last 15 years [12]. As strongly recommended by the Intergovernmental Panel on Climate Change, urgent and deep GHG emissions reductions across all sectors are necessary in order to limit global warming to 1.5 °C [13].

Climate change is a great threat and presents a unique opportunity to protect cardiovascular health in the next decades [14]. In the framework of an integrative, multilevel strategy, cardiologists are essential to better understand the mechanistic link between climate change and cardiovascular disease, to promote multilevel (from the government to individuals) mitigation strategy, identify vulnerable subgroups for more effective treatment of cardiovascular complications [15], and adopt carbon-neutral imaging prescription strategies.

CO_2_ is not strictly a pollutant but a prototype of GHG and an index of the impact of human activities on climate change. CO_2_ is different from an air pollutant since it is present in clean air (at a low concentration of 0.04%), essential for a normal life, emitted in human breath, and not a trigger of acute cardiovascular events. However, it is considered an air pollutant by law for its direct effect on the climate system and indirect effects on health. The new landmark climate law passed by the US Senate in August 2022 amends the Clean Air Act, the country’s air-quality legislation, to define the CO_2_ produced by burning fossil fuels as an “*air pollutant*”. With the new law, Congress has unequivocally told federal agencies to tackle CO_2_ [16]. The Supreme Court ruled, with a 5–4 decision, in 2007 that CO_2_ can be regulated as a pollutant under the Clean Air Act. The Clean Air Act defines “air pollutant” as “any air pollution agent or combination of such agents, including any physical [or] chemical … substance or matter which is emitted into or otherwise enters the ambient air” and “may reasonably be anticipated to endanger public health or welfare.” In its 2007 decision, the Supreme Court correctly recognized that carbon is a “chemical substance or matter” that is “emitted into” the air and “endangers public health” by contributing to rising global temperatures. 

With a new conservative majority, the Supreme Court ruled 6–3 on 30 June 2022, that the Clean Air Act does not give the U.S. Environmental Protection Agency widespread power to regulate CO_2_ emissions. The prevailing opinion reported by Judges of the Supreme Court in 2022 is as follows, “*Now, what is a pollutant? A pollutant is a subject that is harmful to human beings or to animals or plants. CO_2_ is not a pollutant. CO_2_ is not harmful to ordinary things, human beings, or to animals, or plants. It’s actually needed for plant growth. All of us are exhaling CO_2_ right now. So, if it’s a pollutant, we’re all polluting*” [17]. These opinions may be legally valid but are less convincing from a logical, pathophysiological, and medical viewpoint. The CO_2_ increase endangers humans, animals, plants, and the planet. The Climate law passed in August 2022 determined that CO_2_, for all practical and legal purposes, must be treated as an air pollutant, and this approach is more reasonable for physicians.

## 3. The Environmental Footprint of Imaging Examinations 

Marwick and Buonocore evaluated the environmental impact of different cardiac imaging techniques: stress-US, stress-MRI, and stress-SPECT [18]. The impact of MRI and SPECT on human health followed a clear gradient. MRI was associated with the greatest environmental damage. SPECT showed intermediate damage between 4 and 11% of the MRI impact, and US showed the least damage by far, quantified at 0.5–2.0% of the MRI damage. The basis of these differences is largely due to energy consumption, which leads to an estimated CO_2_ emission of 2 to 3 kg per stress-US (approximately 2.2 kg in Italy and 2.9 kg in the USA in 2013) and up to 200–300 kg per stress-MRI study (229 kg in Italy and 302 kg in the USA), as estimated by Braga et al. [19]. With an abdominal scan, the relative inter-imaging differences are similar to those observed for a cardiac stress study, although absolute values are lower. When both the production and use phases are considered, Martin et al. estimated the carbon cost of an abdominal imaging examination at 1.15 kg CO_2_/examination with a US scan, 6.61 kg CO_2_/examination for a CT scan, and 19.72 kg CO_2_/examination with an MRI scan [20]. 

**Figure 1:**
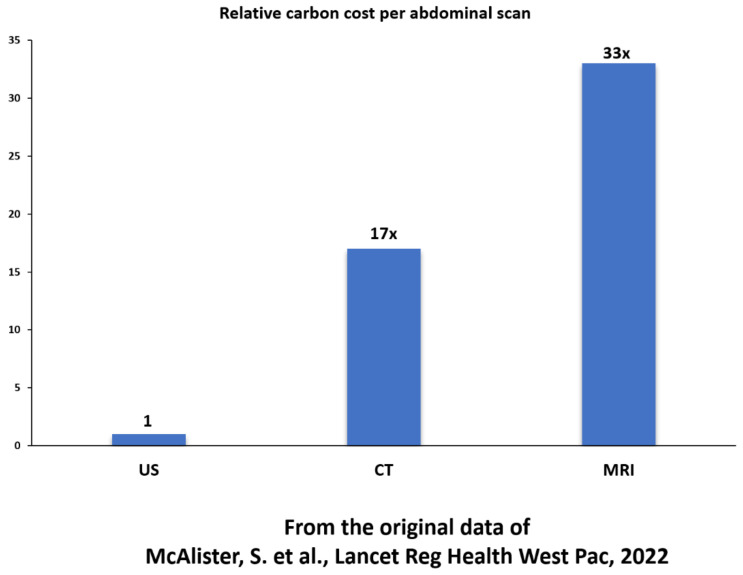
The relative carbon cost (CO_2_ emissions/examination) for an abdominal scan with US (absolute cost 0.5 kg), CT (absolute cost 9.2 kg), and MRI (absolute cost 17.5 kg). From the original data of McAlister et al. [21].

Similar values were reported by Mc Allister et al., with mean CO_2_ emissions/examinations estimated at 0.5 kg for US, 9.2 kg for CT, and 17.5 kg/scan for MRI [21]. The carbon cost changed with the operating mode, the technology employed, the average scan time, the region under investigation, and several other variables, including the utilization rate [22]. In particular, for any given MRI machine and type of scan, the energy cost accounted for 98% of the carbon cost, according to Marwick and Buonocore [18], and it was heavily dependent on the utilization rate. With a modern MRI machine, the average energy cost per exam was estimated at 22.4 kg CO_2_ by Esmaeili et al., with a wide range of values from as low as 48.1 kW/h per patient, with a utilization rate of 90% (262 hospital patients per month), to an almost 10-fold higher value of 399.8 kW/h with a utilization rate of 10% (29 patients per month) [23]. Therefore, any estimate of the absolute carbon cost per exam is subject to many assumptions and local conditions, from energy cost to utilization rate. However, the relative carbon cost is remarkably similar, and there is converging evidence that MRI has the greatest, CT has an intermediate, and the US has the smallest environmental impact (Figure 1). 

**Figure 2:**
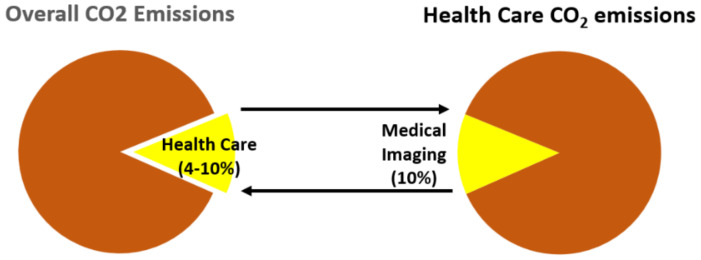
The contribution of healthcare and medical imaging to overall carbon emissions on a planetary scale.

Healthcare CO_2_ emissions are an important part of global emissions. The healthcare carbon footprint contributes significantly to the total national carbon footprint [24,25,26,27], ranging from 10% in the United States in 2016 [25] to an estimated 4% in the United Kingdom [26]. Medical imaging contributes significantly to healthcare’s share of emissions, although the exact contribution is subject to a wide range of assumptions [27,28]. Kouropoulos [29] determined that 0.77% of global emissions in 120 countries were caused by CT and MRI imaging. The study did not include in its estimate emissions from nuclear medicine, invasive fluoroscopy, chest x-rays, and US, which total billions of examinations per year (Figure 2). This estimate is expected to grow by 30% from 2018 to 2030, in line with the expected growth of the global diagnostic imaging market from 2019 to 2024, with an annual growth rate of 5.5%.

The healthcare sector potentially has a key role in climate change mitigation efforts. The environmental footprint is also a cost that, while not immediately taken by the payer as a direct cost, is covered by society as a long-term, downstream, externalized cost all citizens will pay collectively [30].

## 4. Opportunity to Change

The pervasive effects of air pollution and climate change on human health and cardiovascular disease open new challenges but also unprecedented opportunities for cardiovascular disease prevention, diagnosis, and treatment. Greening may affect all phases of medical imaging, from engineering to production to medical end-users. These actions may act efficiently to mitigate the environmental footprint of imaging, thus, targeting more than lowering CO_2_ emissions [31]. For instance, gadolinium-based contrast media for MRI are dispersed in water and may lead to drinking water contamination [32]. The production of costly and toxic radioactive waste with nuclear medicine can be effectively minimized with the development of radiation-free diagnostic methods, as has happened in the last 30 years with stress-US progressively replacing nuclear cardiology for cost and radiation exposure concerns [33]. 

The challenge for the imaging community is to keep high diagnostic standards while reducing the carbon cost of our activities in a proactive role that is in concert with the industry and patients [34]. 

Prescribers and practitioners “*must urgently consider the role of imaging in climate change and mitigate imaging’s harmful environmental impact*”, as recently stated in a 2021 call to action from the President of the American College of Radiology [35]. When the diagnostic information provided by different imaging techniques is similar, low-carbon testing is preferential over high-carbon testing. Inappropriate testing represents about 50% of current testing volumes and should be avoided [36], especially imaging techniques associated with high carbon emissions.

There are some data on CO_2_ emissions/examinations for US, CT, and MRI and some relatively old data on SPECT. However, the carbon footprint information is completely missing for stress-PET, which is widely used in cardiac stress testing with a rising utilization rate due to lower radiation exposure than stress-SPECT. It would be important to have more detailed and specific data according to the technology used, operating mode (rest or stress), use of contrast (for echo or MRI), and drugs employed during the study to generate an exhaustive catalog of carbon costs to be associated with each imaging procedure. This information will increase the awareness of doctors and patients and make carbon-neutral choices easier. 

For instance, magnetic resonance will greatly benefit from industrial developments shifting the focus from machines with high-intensity magnetic fields (3 Tesla or even 6 Tesla) requiring high energy consumption to an energy-sparing strategy, leading to the diffusion of new technologies aimed at obtaining the same image quality with lower intensity fields and much less energy expenditure [37]. 

## 5. The Road to Environmental Sustainability in Medical Imaging

Clinicians need simple rules to address the complex issue of environmental impact. We need more awareness, information, and data to drive a change in time-honored prescription patterns, feeding legitimate vested interests. There is an opportunity to follow in the footsteps of social marketing and scientific society campaigns, such as Choosing Wisely and Image Gently, to reduce the unacceptable rate of inappropriate imaging examinations and unjustified or unoptimized radiological examinations. These campaigns targeted patients and doctors, reducing inappropriate testing and dramatically lowering of the radiation dose per exam through the combined effort of industry, scientists, clinicians, and patient organizations [38,39]. Any effort toward environmental sustainability will focus on three stakeholders: doctors, industry, and patients. 

A recent survey in Italy documented that 93% of doctors believe that the emissions of CO_2_ per examination should become an important factor in decision-making for cardiac imaging [40]. The industry is also important due to its efforts in creating modern and improved medical imaging devices with less carbon cost but no loss in resolution [41]. The third stakeholder is the patient. A survey in the United Kingdom showed that 92% of patients believe sustainability in healthcare operations is important [42]. Ideally, the informed consent paperwork should include the radiologic exposure in multiples of chest X-rays and the carbon cost in kg of CO_2_ emissions compared to standard activities familiar to the patient, such as driving a car for a certain number of kilometers. Using the updated estimates from Mc Allister et al., an abdominal US scan (0.5 kg of CO_2_ emissions) corresponds to driving a modern car for 4 km, a CT (9.2 kg of CO_2_ emissions) for 76 km, and a resting MRI (17.5 kg of CO_2_ emissions) for 145 km [21]. This is a way to inform and explain to patients and doctors what they often ignore regarding the environmental impact of medical and, specifically, imaging procedures. 

Choosing Wisely could be a model to reach the ambitious goal of better environmental sustainability of medical imaging in the next decade. In the meantime, some simple rules driven by the available data and common sense can help in everyday practice for a green transition without lowering the current standard of health care in cardiac imaging:

1. As prescribers or practitioners, we need to know what our carbon impact is (the exact environmental cost of each imaging examination);
2. There is no right or wrong absolute value of carbon cost, but certainly, the wrong value is the one we ignore;
3. Through its effects on climate change, carbon is a cost taken up by society;
4. Through its effects on climate change, carbon is a health risk taken up by the entire population, including those unexposed to testing;
5. Like radiation, carbon cost should always be justified;
6. Like radiation, carbon cost should always be optimized;
7. Like radiation, the responsibility for inappropriate carbon costs should be that of both the prescriber and the practitioner;
8. If we include carbon cost in our imaging cost-benefit balance, the industry will start on the road to achieving the same image quality with less carbon cost;
9. Carbon is important in all fields of the economy, from housing to energy and transportation, and also in medicine to preserve the current standard of care and environmental integrity for future generations;
10. Prescribing an imaging test is a medical and social act [43].

**Figure 3:**
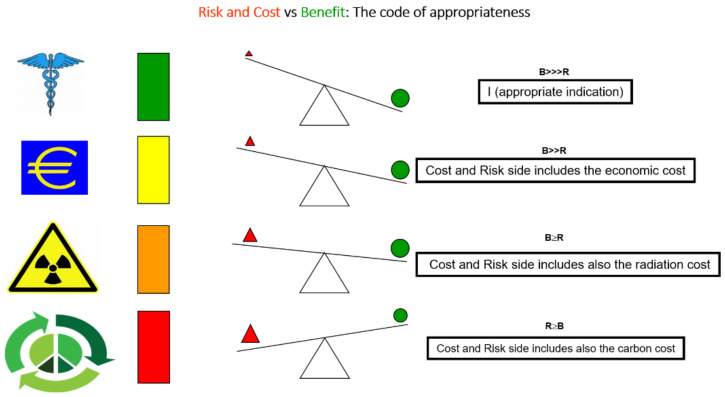
The cost-benefit assessment in medical imaging (first row) includes the medical benefit (such as diagnostic accuracy) balanced with the acute risks, such as the occurrence of myocardial infarction during stress. Over the last two decades, a more comprehensive assessment was introduced. Integrating on the cost side is the economic cost (second row), the radiological risk linearly related to the long-term risk of cancer (third row), and now, the environmental or carbon cost related to the detrimental effect on the planet (last row).

The cost-benefit assessment should include immediate cost, radiation risk, and carbon cost (Figure 3). 

It is unrealistic to achieve the goal of sustainability without, or even against, the medical imaging community. Conversely, the cardiology and cardiac imaging community cannot remain neutral in this debate. Environmental sustainability in cardiology and cardiac imaging is essential in the quest for sustainability in healthcare [44].

## 6. Conclusions

Human health is one of the earliest biosensors of climate change, and cardiovascular disease increases in frequency and severity with climate change. Doctors are fearful of climate change, but also a part of the problem since healthcare contributes significantly to the overall carbon footprint [45]. Cardiac imaging immensely improved the quality of cardiology care, but it is also a recognized source of population damage through radiation exposure and environmental damage through CO_2_ emissions. The cardiology and cardiac imaging communities can have a proactive role in the decarbonization of imaging, in teamwork with industry and patients, and as gatekeepers to improve imaging quality and cost-effectiveness. The Choosing Wisely campaign celebrated its 10th birthday in 2022 and has taught patients and doctors that overuse is an equity issue and that avoiding overuse can protect patient safety [46]. The Green Heart initiative will eventually push Choosing Wisely’s gratifying experience of promoting appropriateness in imaging further to include the economic and radiological cost and the carbon cost of medical imaging in decision-making. Avoiding medical imaging overuse can also protect planet safety [47].

## Data Availability

The data presented in this study are available on request from the corresponding author. The data are not publicly available due to privacy law.
